# Prevalence of Hypertension and Its Associated Risk Factors Among Adults Attending Medical Outpatient Clinics at Ibn Sina General Hospital Authority in Mukalla City, Yemen

**DOI:** 10.7759/cureus.60540

**Published:** 2024-05-18

**Authors:** Lotfi Bin Dahman, Amir M Al-Awbathani, Abdulrhman A Bawazir, Ahmed S Al-Awbathani, Hussain A Alhabshey, Halima O Saad, Noran A Ahmed

**Affiliations:** 1 Clinical Biochemistry, Hadhramout University, Mukalla, YEM; 2 College of Medicine, Hadhramout University, Mukalla, YEM

**Keywords:** cross-sectional, mukalla city, risk factor, blood pressure, hypertension

## Abstract

Background: Hypertension (HTN) is the most generally acknowledged modifiable risk factor for cardiovascular disease, cerebrovascular disease, and end-stage renal disease. Accordingly, the World Health Organization has listed HTN as the third greatest cause of death globally.

Objectives: The objective of this study was to assess the prevalence of HTN and its associated risk factors among adults attending medical clinics at Ibn Sina Hospital Authority in Mukalla City, Yemen.

Methods: A cross-sectional descriptive survey was conducted using a self-administered questionnaire applied to 384 male and female adults aged ≥18 years attending Ibn Sina General Hospital Authority outpatient clinics in Mukalla City, Yemen, between December 2022 and May 2023. The participant’s body weight, height, and waist circumference were measured. The data were analyzed using Statistical Package for the Social Sciences (IBM SPSS Statistics for Windows, IBM Corp., Version 25.0, Armonk, NY). P values of <0.05 were considered statistically significant.

Results: Among the 384 participants, 20.5% had HTN, and the remaining (79.5%) did not have HTN, with a substantial proportion (47.2%) reporting a positive family history of HTN. Diabetes mellitus was present in 16.1% of the participants, whereas dyslipidemia and other chronic diseases were reported by 9.3% and 15.8% of the participants, respectively. A total of 75.6% of the participants had never smoked, and 11.7% were past smokers. More than half of the participants (57.29%) had never chewed khat, 20.57% were former khat chewers, and 22.14% were currently chewing khat. Nutritional status, as indicated by body mass index, showed that 29.8% were overweight.

Conclusions: HTN was found to be prevalent among the study participants. However, the respondents’ awareness of the problem and the overall control rates were very low. Certain factors, such as family history of HTN, diabetes mellitus, and high body mass index, were found to be associated with HTN. Therefore, intervention measures are warranted emphasizing modifiable risk factors to prevent HTN.

## Introduction

Hypertension (HTN), which is a significant public health issue on a global scale, is the most generally acknowledged modifiable risk factor for cardiovascular disease (CVD), cerebrovascular disease, and end-stage renal disease [1,2]. Accordingly, the World Health Organization (WHO) has listed HTN as the third highest cause of death globally, accounting for one in eight of all fatalities [3]. Along with HTN, abdominal obesity, dyslipidemia, and insulin resistance are also common risk factors for CVD [4].

The impact of the HTN epidemic has been felt by all nations, regardless of economic status. Nearly one billion individuals worldwide have HTN, with two-thirds of them living in low-income countries [5]. Approximately 10 million individuals die each year from HTN, which affects an estimated 1.3 billion people globally [6]. As a result of multiple factors, including economic growth and population aging, the prevalence of HTN has increased over the past few decades worldwide, even in developing countries [7]. The prevalence of HTN in adults can be influenced by variations in study protocols, genetic background, and environmental factors such as food intake and physical activity [8].

A new set of guidelines for the prevention and management of HTN was recently outlined in the Seventh Report of the Joint National Committee on Prevention, Detection, Evaluation, and Treatment of High Blood Pressure (JNC-7). The JNC-7 report defines normal blood pressure as having a systolic blood pressure (SBP) of <120 mmHg and a diastolic blood pressure (DBP) of <80 mmHg; whereas pre-HTN is defined as having an SBP of 120-139 mmHg or a DBP of 80-90 mmHg [9]. HTN is considered present when blood pressure is taken on two different days with systolic and/or diastolic readings both greater than 140 mmHg [10,11].

HTN prevalence varies widely around the world, ranging from 3.4% to 78%, with South Africa having the greatest frequency and rural India having the lowest, according to an analysis of data from six countries [12]; HTN prevalence was reported to be 49.39% in Malaysia. Age, household income, body mass index (BMI), and diabetes are all strongly associated with HTN [13]. In Turkey, pre-HTN showed a prevalence of 14.5%, whereas that of HTN was 44.0%. The age range of 60-69 years exhibited the highest prevalence of HTN, which increased with aging. Interestingly, HTN is inversely correlated with the amount of education, current cigarette usage, and physical activity; and positively correlated with marital status, parity, and quitting smoking [14]. In Nepal, the prevalence of HTN was 22.4% overall (males: 32.7% and females: 15.3%). HTN prevalence significantly increases with age, as indicated by age-specific frequencies of 8-35% [15]. In Ethiopia, the prevalence of HTN was 21.2% among the general population; where age, occupation, wealth status, consuming vegetables and animal fat, BMI family history of HTN, and diabetes were associated with the presence of HTN at 95% confidence intervals (CIs) [16]. In a 2013 study in Saudi Arabia, 917,188 (7.1%) Saudis reported a diagnosis of HTN; age, sex, and previous diabetes and high cholesterol diagnoses were linked to HTN [17].

As there has been no recent study conducted and published on adults regarding HTN in Yemen, and because of the different highly associated risk factors that are practiced in the study region, it is very important to use descriptive studies when no previous studies are available. Therefore, in this study, we aim to measure the prevalence of HTN and its associated risk factors among adults attending Ibn Sina General Hospital Authority outpatient clinics in Mukalla City, Yemen.

## Materials and methods

Study design

This study adopted a cross-sectional descriptive survey design. The study was performed by a group of medical students at Hadhramout University, Mukalla, Yemen.

Study setting and duration

The study was conducted at Ibn Sina General Hospital Authority in Mukalla City, Yemen. Ibn Sina General Hospital Authority is a hospital with a usable area of 37,500 m^2^ and a clinical capacity of 300 beds, providing services to the governorates of Hadhramout, Shabwa, and Al-Mahrah, as well as the island of Socotra and some villages of Hadhramout. In general, it is estimated that the hospital provides services to 33% of the total area of the eastern region of Yemen. The study was conducted between December 2022 and May 2023.

Study population

The study population comprised all adults attending Ibn Sina General Hospital Authority outpatient clinics in Mukalla City at the time of data collection. The participants were considered hypertensive when they were documented to have an increase in blood pressure.

Inclusion criteria

The following inclusion criteria were used for the selection of participants: adults attending outpatient clinics in Ibn Sina General Hospital Authority in Mukalla City at the time of the study who were either diagnosed with HTN or not. According to the National Institutes of Health (NIH), an adult is a person ≥18 years of age unless national law delimits an earlier age [18].

Exclusion criteria

Participants were excluded from the study if they met any of the following criteria: attendants who are not considered adults according to the aforementioned NIH definition, those who have intellectual disability, and all patients with severe illnesses, acute life-threatening conditions, or severe injury, including patients with head injuries.

Sample size estimation

The sample size was calculated using the WHO formula for estimating the sample size. The level of certainty was 1.96, the proportion of the characteristics in the population was 50%, and the precision or allowable error was 5%.

Sampling method

Selection of the participants was done by convenience sampling among adults who visited all Ibn Sina General Hospital Authority outpatient clinics (excluding the psychiatry clinic due to insufficient data) during the study period. We chose the number of participants from each clinic by measuring the flow rate per clinic in August, September, and October of the year 2022 to ensure that every outpatient clinic visitor had an equal chance to participate in the study (Table 1). The sample size was distributed proportionally among the 12 available outpatient clinics in Ibn Sina General Hospital Authority according to the number of adults visiting each clinic (i.e., the flow rate in the last three months) (Table 2). The total number of adults attending the outpatient clinics from August to October of 2022 was 7883.

**Table 1:** Flow rate of available outpatient clinics from August to October 2022

Clinics	August	September	October	Total
Orthopedic	360	312	445	1117
Dental	10	0	30	40
Dermatology	160	142	176	478
Ophthalmology	116	102	74	292
Internal medicine	543	382	500	1425
Neurology	252	244	423	919
General surgery	287	303	289	879
General	192	140	211	543
Ear, nose and throat	289	280	446	1015
Cardiology	18	48	36	102
Urology	235	216	225	676
Endocrine	92	119	186	397

**Table 2:** Distribution of the included sample population

Clinics	Number of patient in each clinic	Percentage	Sample size
Orthopedic	1117	14.16%	55
Dental	40	0.50%	2
Dermatology	478	6.06%	23
Ophthalmology	292	3.70%	14
Internal medicine	1425	18.07%	70
Neurology	919	11.68%	45
General surgery	879	11.15%	43
General	543	6.88%	26
Ear, nose and throat	1015	12.89%	49
Cardiology	102	1.29%	5
Urology	676	8.57%	33
Endocrine	397	5.05%	19

Data collection and tools

A structured questionnaire with close-ended questions was designed by using the WHO STEPwise approach to non-communicable disease risk factor surveillance [19]. The questionnaire was developed in English and then translated into Arabic by experts. The questionnaire was designed to have two main parts: part one included questions about socio-demographic information, and part two included questions about risk factors associated with HTN. In addition, a weight scale, a stadiometer, and a measuring tape were used to measure weight, height, and waist circumference, respectively [20].

Data analysis

Data entry and statistical analysis were done using Statistical Package for the Social Sciences (IBM SPSS Statistics for Windows, IBM Corp., Version 25.0, Armonk, NY). Data are presented using descriptive statistics in the form of frequencies and percentages. Binary regression was used to find associations between variables. Statistical significance was considered at p<0.05.

Ethical considerations

Everyone who took part in the study provided oral informed consent. The revised Declaration of Helsinki served as the foundation for the present study. In addition, approval for this study was obtained from Hadhramaut University, College of Medicine, Department of Community Medicine (approval number: CM/REC/4/2023), and from the participants. The participants were assured that they would remain anonymous and that their data would remain confidential.

## Results

Finally, our cross-sectional study included 384 participants. As shown in Table 3, 63.7% of the participants were male, and 36.3% were female. The age distribution was diverse, with 33.6% aged 18-29, 24.4% aged 30-39, 17.1% aged 40-49, and 24.9% aged ≥50. The majority of participants were married (65%). Although the educational level varied, it was clear that the majority (31.3%) of participants were at the primary school level. Employment status was diverse, with 28.8% of participants being government employees and 21.5% being self-employed. The monthly income distribution showed that 39.6% of participants earned <50,000 Yemeni Riyal, and 20.8% earned >100,000 Yemeni Riyal.

**Table 3:** Socio-demographic characteristics of the study participants (n= 384)

Items	Variable	Frequency	Percentage %
Sex	Male	246	63.7
	Female	138	36.3
Age (years)	18-29	128	33.6
	30-39	94	24.4
	40-49	66	17.1
	≥50	96	24.9
Marital status	Never married	106	27.5
	Currently married	251	65
	Divorced	10	2.6
	Widowed	15	4.4
	Nonresponse	2	0.5
Monthly income (Yemeni Riyal)	<50000	152	39.6
	50000-100000	152	39.6
	>100000	80	20.8
Level of education	Illiterate	69	17.9
	Primary school level	121	31.3
	Secondary school level	90	23.3
	Diploma	46	11.9
	University graduate (bachelor's degree) and above	58	15.6
Employment	Government employee	111	28.8
	Self-employed	83	21.5
	Student	43	11.1
	Housewife	76	19.7
	Daily laborer	21	5.4
	Merchant	3	0.8
	Unemployed (able to work)	24	6.2
	Unemployed (unable to work)	7	1.8
	Retired	16	4.7

Among the participants, 20.5% had HTN, and 47.2% reported a positive family history of HTN. Diabetes mellitus was present in 16.1% of participants, whereas dyslipidemia and other chronic diseases were present in 9.3% and 15.8% of participants, respectively (Table 4).

**Table 4:** Biochemical measures of the study participants (n= 384)

Item	Variable	Frequency	Percentage %
Do you have hypertension?	Yes	79	20.5
	No	305	79.5
Do you have a positive family history of hypertension?	Yes	182	47.2
	No	202	52.8
Do you have diabetes mellitus?	Yes	62	16.1
	No	322	83.9
Do you have dyslipidemia?	Yes	36	9.3
	No	348	90.7
Diagnosis of any chronic hypertension?	Yes	59	15.8
	No	325	84.2

Regarding behavioral risk factors, 75.6% of participants had never smoked, and 57.29% had never chewed khat. Dietary habits showed that 59.6% consumed fruits one to three days per week, and 57.3% consumed vegetables one to three days per week. Most participants (63.8%) perceived their salt intake as adequate (Table 5).

**Table 5:** Behavioral measures of study participants (n=384)

Items	Variable	Frequency	Percentage %
Smoking status	Never smoked	292	75.6
	Past smoker	43	11.7
	Current smoker	49	12.7
Khat chewing status	Never chewed	220	57.29
	Past chewer	79	20.57
	Current chewer	85	22.14
Fruit servings consumed in days per week	None	47	12.2
	1-3 days	230	59.6
	4-7 days	76	20.2
	Do not know	31	8
Vegetable servings consumed in days per week	None	42	10.9
	1-3 days	219	57.3
	4-7 days	97	25.1
	Do not know	26	6.7
How much salt do you think you consume?	Far too much	16	4.1
	Too much	44	11.4
	Just the right amount	244	63.8
	Too little	40	10.4
	Far too little	38	9.8
	Do not know	2	0.5

Nutritional status, as indicated by BMI, showed that 10.1% of the participants were underweight, 36.2% had a normal BMI, 29.8% were overweight, 19.2% were classified as having class I obesity, and 4.7% had a BMI exceeding 35. Waist circumference measurements demonstrated that 28.8% of male participants had a waist circumference of <90 cm, whereas 36.2% had a waist circumference of ≥90 cm. Among the female participants, 12.7% had a waist circumference of <80 cm, and 22.3% had a waist circumference of ≥80 cm (Table 6).

**Table 6:** Nutritional status of study participants (n=384)

Item		Variable	Frequency	Percentage %
Body mass index (kg/m^2^)		<18.5	39	10.1
		18.5-24.9	138	36.2
		25.0-29.9	115	29.8
		30.0-34.9	74	19.2
		≥35	18	4.7
Waist circumference (cm)	Men	<90	111	28.8 (44.57% of men)
		≥90	138	36.2 (55.42% of men)
	Women	<80	49	12.7 (36.29% of women)
		≥80	86	22.3 (63.70% of women)

Based on our results, we found a significant statistical association between the presence of HTN and some variables, whereas others showed no association. For example, increased age, diabetes mellitus, dyslipidemia, history of smoking, and BMIs of 30.0-34.9 showed a statistically significant association with HTN, as their p-values were <0.05. On the other hand, sex, marital status, educational status, employment status, monthly income, family history of HTN, khat chewing status, fruit and vegetable consumption, salt consumption, physical activity, BMIs except 30.0-34.9, and waist circumference did not show a statistically significant association with HTN, as their p*-*values were >0.05. Therefore, we can say that the association between HTN and some variables was supported by the logistic regression analysis, whereas that of other variables was not supported (Tables 7-10).

**Table 7:** Multivariate analysis of the socio-demographic data in association with hypertension *Considered statistically significant; n: number; Ref: reference; OR: odd ratio; CI: confidence interval

Variable		Hypertension			OR (95% CI)	*P*-value
		Yes n(%)	No n(%)	Total n(%)		
Sex	Male	52(21.13)	194(78.87)	246(100)	Ref	
	Female	27(19.56)	111(80.44)	138(100)	0.643(0.268-1.543)	0.323
Age (years)	18-29	4(3.23)	124(96.77)	128(100)	Ref	
	30-39	17(18.08)	77(81.92)	94(100)	0.163(0.046-0.583)	<0.005*
	40-49	16(24.25)	50(75.75)	66(100)	0.102(0.026-0.404)	<0.001*
	≥50	42(43.75)	54(56.25)	96(100)	0.053(0.014-0.200)	<0.001*
Marital status	Never married	8(7.55)	98(92.45)	106(100)	Ref	
	Currently married	61(24.31)	190(75.69)	251(100)	0.944(0.343-2.599)	0.912
	Divorced	3(30)	7(70)	10(100)	0.676(0.118-3.874)	0.661
	Widowed	6(40)	9(60)	15(100)	0.553(0.112-2.737)	0.468
	Refuse to answer	1(50)	1(50)	2(100)	0.046(0.002-1.342)	0.074
Educational status	Illiterate	18(26.09)	51(73.91)	69(100)	Ref	
	Primary school level	29(23.96)	92(76.04)	121(100)	0.759(0.326-1.767)	0.522
	Secondary school level	19(21.12)	71(78.88)	90(100)	0.843(0.318-2.234)	0.732
	Diploma	8(17.40)	38(82.60)	46(100)	0.894(0.274-2.915)	0.853
	Bachelor and above	5(8.63)	53(91.37)	58(100)	1.625(0.416-6.353)	0.485
Employment status	Government employee	27(24.33)	84(75.67)	111(100)	Ref	
	Self-employed	14(16.86)	69(83.14)	83(100)	1.079(0.476-2.447)	0.855
	Student	1(2.33)	42(97.67)	43(100)	2.341(0.211-26.036)	0.489
	Housewife	14(18.43)	62(81.57)	76(100)	2.734(0.875-8.540)	0.084
	Daily labor	4(19.05)	17(80.95)	21(100)	1.082(0.291-4.019)	0.906
	Merchant	1(33.34)	2(66.66)	3(100)	0.351(0.019-6.461)	0.481
	Unemployed (able to work)	5(20.84)	19(79.16)	24(100)	1.250(0.355-4.407)	0.729
	Unemployed (unable to work)	4(57.15)	3(42.85)	7(100)	0.592(0.104-3.372)	0.555
	Retired	9(56.25)	7(43.75)	16(100)	0.508(0.154-1.675)	0.266
Monthly income (Yemeni Riyal)	<50,000	28(18.43)	124(81.57)	152(100)	Ref	
	50,000-100,00	36(23.68)	116(76.32)	152(100)	1.054(0.550-2.020)	0.875
	>100,000	15(18.75)	65(81.25)	80(100)	1.118(0.482-2.594)	0.795

**Table 8:** Multivariate analysis of the biochemical data in association with hypertension *Considered statistically significant; n: number; Ref: reference; OD: odd ratio; CI: confidence interval; HTN: hypertension

Variable		Hypertension				OR (95% CI)	*P*-value
		Yes n(%)	No n(%)	Total n(%)			
Positive family history of HTN	Yes	48(26.37)	134(73.63)		182(100)	Ref	
	No	31(15.35)	171(84.65)		202(100)	0.588(0.336-1.029)	0.063
Diabetes mellitus	Yes	35(56.45)	27(43.55)		62(100)	Ref	
	No	44(13.66)	278(86.34)		322(100)	0.174(0.089-0.337)	<0.001*
Dyslipidemia	Yes	19(52.77)	17(47.23)		36(100)	Ref	
	No	60(17.25)	288(82.75)		348(100)	0.403(0.176-0.923)	0.032*
Any chronic diseases	Yes	26(44.06)	33(55.94)		59(100)	Ref	
	No	53(16.30)	272(83.70)		325(100)	0.628(0.306-1.289)	0.205

**Table 9:** Multivariate analysis of the behavioral data in association with hypertension *Considered statistically significant; n: number; Ref: reference; OR: odd ratio; CI: confidence interval

Variable		Hypertension			OR (95% CI)	*P*-value
		Yes n(%)	No n(%)	Total n(%)		
Smoking status	Never smoked	50(17.13)	242(82.87)	292(100)	Ref	
	Past smoker	17(39.53)	26(60.46)	43(100)	0.307(0.151-0.626)	<0.001*
	Current smoker	12(24.48)	37(75.52)	49(100)	0.611(0.282-1.323)	0.212
Khat chewing status	Never chewed	54(24.55)	166(75.45)	220(100)	Ref	
	Past chewer	16(21.92)	57(78.08)	73(100)	0.799(0.449-1.422)	0.445
	Current chewer	9(9.90)	82(90.10)	91(100)	1.08(0.786-1.361)	0.233
Fruit consumption/week	None	12(25.54)	35(74.46)	47(100)	Ref	
	1-3 days	43(18.70)	187(81.30)	230(100)	2.397(0.879-6.533)	0.088
	4-7 days	21(27.64)	55(72.36)	76(100)	1.240(0.395-3.892)	0.712
	Do not know	3(9.6)	28(90.40)	31(100)	1.981(0.360-10.907)	0.423
Vegetable consumption/week	None	8(19.05)	34(80.95)	42(100)	Ref	
	1-3 days	48(21.92)	171(78.08)	219(100)	0.456(0.147-1.414)	0.174
	4-7 days	22(22.68)	75(77.32)	97(100)	0.594(0.169-2.088)	0.416
	Do not know	1(3.85)	25(96.15)	26(100)	3.556(0.285-44.314)	0.324
Salt consumption	Far too much	1(6.25)	15(93.75)	16(100)	Ref	
	Too much	14(31.81)	30(68.19)	44(100)	0.140(0.017-1.184)	0.071
	The right amount	42(17.22)	202(82.78)	244(100)	0.319(0.041-2.508)	0.278
	Too little	7(17.5)	33(82.5)	40(100)	0.288(0.032-2.599)	0.267
	Far too little	15(39.47)	23(60.53)	38(100)	0.101(0.012-0.852)	0.035*
	Do not know	0(0)	2(100)	2(100)	161495176.0(000)	

**Table 10:** Multivariate analysis of the nutritional data in association with hypertension *Considered statistically significant; n: number; Ref: reference; OR: odd ratio; CI: confidence interval

Variable			Hypertension			OR (95% CI)	*P*-value
			Yes n(%)	No n(%)	Total n(%)		
Body mass index (kg/m^2^)	<18.5		2(5.12)	37(94.88)	39(100)	Ref	
	18.5-24.9		22(15.95)	116(84.05)	138(100)	0.321(0.071-1.447)	0.139
	25.0-29.9		28(24.35)	87(75.65)	115(100)	0.230(0.049-1.088)	0.064
	30.0-34.9		24(32.44)	50(67.56)	74(100)	0.154(0.031-0.760)	0.022*
	≥35		3(16.66)	15(83.34)	18(100)	0.381(0.053-2.721)	0.336
Waist circumference (cm)	Men	<90	13(11.72)	98(88.28)	111(100)	Ref	
		≥90	39(28.26)	99(71.74)	138(100)	0.543(0.243-1.217)	0.138
	Women	<80	9(18.36)	40(81.64)	49(100)	Ref	
		≥80	18(20.94)	68(79.06)	86(100)	0.822(0.335-2.015)	0.668

## Discussion

HTN, as the most common cardiovascular disorder, is now regarded as a major public health problem. Many people may not be aware that they have HTN until they develop complications such as heart attack, stroke, or kidney failure. Therefore, early detection, diagnosis, and management of HTN are critical in preventing or delaying the onset of these complications [21].

As no recent study has identified the prevalence of HTN in Yemen, this study was conducted to identify the magnitude of the problem of HTN among individuals attending medical outpatient clinics, who are likely to have underlying health conditions. By identifying the risk factors associated with HTN, both the public and healthcare providers can develop policies and targeted interventions to prevent or manage HTN effectively [22]. In our community-based cross-sectional study, which included 384 individuals aged ≥18 years, HTN had a prevalence of 20.5%, which is lower than prior studies in Nepal (22.4%) [15] and Kurdistan, Iraq (25.3%) [23], and higher than that recently reported in one study of four Sudanese states (Khartoum, Gezira, Blue Nile, and Kassala; 15.9%) [24] and in another hospital-based study conducted in Ethiopia (10.55%) [25].

This study revealed a widespread prevalence of various risk factors among participants. HTN prevalence was significantly higher in males than in females. Similarly, numerous studies have found that more men have HTN than women [26-28]. One proposed explanation for the gender gap in HTN prevalence is a combination of biological sex differences and behavioral risk factors such as smoking, alcohol use, and physical activity. We hypothesize that abstaining from chewing khat and smoking are two preventive factors against HTN in women. In addition, as women have been found to be more interested in using healthcare services and are more likely to report ill health, they are more likely to have better health [29,30]. HTN was more prevalent in subjects aged ≥50 years. Other studies revealed similar findings, in which increased age was positively correlated with HTN [30,14]. More than half (56.45%) of the participants with HTN also had diabetes mellitus. Forty-eight of the participants with HTN had a positive family history of HTN, which indicated a strong correlation between the two. Approximately 73.43% of participants with HTN had a BMI of ≥25. Other studies have also reported a direct relationship between high BMI and an increasing rate of HTN [30,31]. Our results did not show a significant association between smoking, khat chewing, and HTN. Our study also revealed that more than half of the participants with HTN did not consume enough fruits and vegetables.

We believe that the findings of our study will contribute to the efforts made to uncover the burden of HTN in our community. One of the strengths of this study is that its topic is not very similar to those of other studies in Mukalla, Yemen, making it of scientific value; furthermore, the participants from each clinic were chosen without any type of bias, the data were collected using a questionnaire, and the sample size was calculated using a specific formula. However, there are certain limitations of this study worth mentioning. First, as this was a cross-sectional study with a relatively small sample size, it is difficult to generalize the findings for the country as a whole. Besides, assessments of behavioral risk factors such as smoking and khat chewing were based on only the history of use of these substances without assessment of the amount and duration of use. As a result, a cause-and-effect relationship could not be established.

## Conclusions

HTN was found to be prevalent among our study population. Certain factors, such as family history of HTN, diabetes, and high BMI were found to be associated with HTN. The findings of this study clearly show that HTN is becoming a serious public health concern in our community. Health education and other measures should emphasize the prevention, early detection, and treatment of HTN. Furthermore, researchers and healthcare providers should work to address the overall burden of HTN.
